# Crystallographic Orientation Dependence of Surface Segregation and Alloying on PdCu Catalysts for CO_2_ Hydrogenation

**DOI:** 10.1021/acs.jpclett.1c00179

**Published:** 2021-03-09

**Authors:** Lukas Pielsticker, Ioannis Zegkinoglou, Zhong-Kang Han, Juan J. Navarro, Sebastian Kunze, Osman Karslıoğlu, Sergey V. Levchenko, Beatriz Roldan Cuenya

**Affiliations:** † Faculty of Physics and Astronomy, Ruhr University Bochum, 44780 Bochum, Germany; ‡ Center for Energy Science and Technology, Skolkovo Institute of Science and Technology, Moscow 121205, Russia; § Department of Interface Science, Fritz-Haber Institute of the Max Planck Society, Berlin 14195, Germany

## Abstract

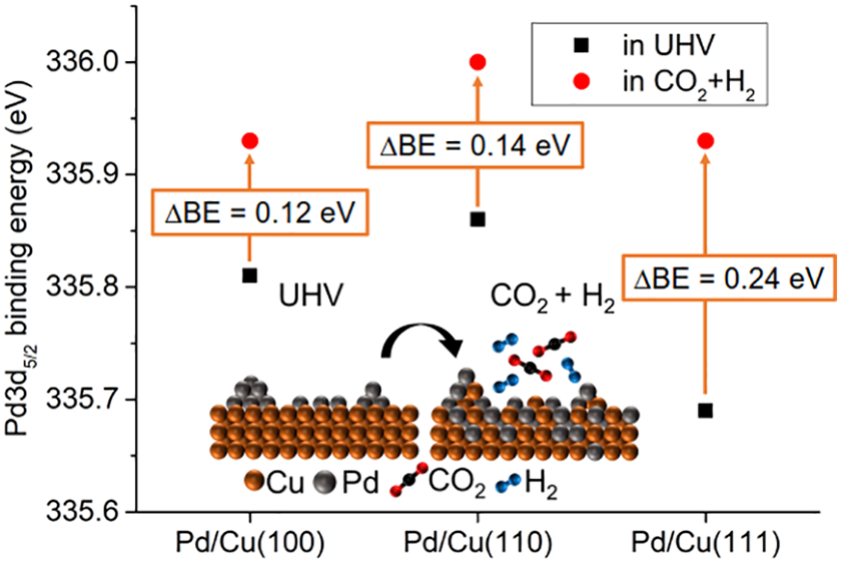

The influence of the crystallographic orientation on surface segregation and alloy formation in model PdCu methanol synthesis catalysts was investigated *in situ* using near-ambient pressure X-ray photoelectron spectroscopy under CO_2_ hydrogenation conditions. Combined with scanning tunneling microscopy and density functional theory calculations, the study showed that submonolayers of Pd undergo spontaneous alloy formation on Cu(110) and Cu(100) surfaces in vacuum, whereas they do not form an alloy on Cu(111). Upon heating in H_2_, inward diffusion of Pd into the Cu lattice is favored, facilitating alloying on all Cu surfaces. Under CO_2_ hydrogenation reaction conditions, the alloying trend becomes stronger, promoted by the reaction intermediate HCOO*, especially on Pd/Cu(110). This work demonstrates that surface alloying may be a key factor in the enhancement of the catalytic activity of PdCu catalysts as compared to their monometallic counterparts. Furthermore, it sheds light on the hydrogen activation mechanism during catalytic hydrogenation on copper-based catalysts.

Methanol (CH_3_OH), an important base chemical and platform molecule for C1 chemistry,^1^ is commercially produced over a Cu/ZnO/Al_2_O_3_ catalyst at high pressures (50–120 bar) and moderate temperatures (200–300 °C)^2^ using a synthesis gas feed (syngas) consisting of varying amounts of H_2_, CO, and CO_2_.^3^ In light of the environmental challenges related to the greenhouse gas CO_2_, methanol production via direct hydrogenation of pure CO_2_ with H_2_ would be beneficial as compared to the CO-heavy process involving syngas.^4−6^ High selectivities toward methanol from CO_2_ and H_2_ can in principle be achieved using conventional Cu/ZnO-based catalysts.^7^ However, the absence of CO in the gas feed results in a decreased reaction rate and an unfavorable shift of the thermodynamic equilibrium, requiring higher reaction temperatures. In addition, the water which is formed as a byproduct cannot be removed in this case through the water–gas shift reaction. This results in kinetic inhibition of the reaction and premature deactivation of the Cu/ZnO catalysts.^8−11^

In an effort to develop highly active and selective catalysts for CO_2_ hydrogenation to methanol, bimetallic systems are particularly interesting because their catalytic properties can be fine-tuned by modifying the active sites and hence the binding strength of the reaction intermediates.^12,13^ Several studies have shown that bimetallic PdCu catalysts exhibit increased methanol production as compared to monometallic systems. It was shown that adding even small amounts of Pd to Cu-based catalysts exceedingly increases the activity in methanol synthesis from CO_2_ and H_2_.^14−19^ Similar observations have been made for Cu–Pd systems employed as catalysts in the electrochemical CO_2_ reduction.^20^ The enhanced activity of PdCu catalysts compared to their monometallic Cu counterparts was explained mainly by an increase in the reactivity of active Cu sites. The dispersion and surface concentration of Cu was found to increase due to the interaction with highly dispersed Pd, with Cu sites being more reduced due to electron donation from Pd.^14^

Whenever a bimetallic catalyst is employed, variations of the elemental surface composition occurring during the reaction are of utmost importance for the catalytic activity. Since the surface structure of bimetallic catalysts is determined by alloying and segregation of the constituent metals, it is necessary to understand the different parameters governing these effects. For bimetallic catalysts containing Cu, we have recently shown that the initial oxidation state, the gas feed composition, and the presence of reaction intermediates play a significant role in determining the surface composition under reaction conditions and, therefore, the catalytic activity.^12,13^

Here, we report a combined near-ambient pressure X-ray photoelectron spectroscopy (NAP-XPS) and density functional theory (DFT) study on a model system consisting of Pd nanoislands grown via evaporation on Cu single crystals. The surface composition after preparation in vacuum, in a reductive hydrogen environment, and under *operando* CO_2_ hydrogenation conditions was systematically investigated. Changes in the Pd 3d binding energy observed in the XPS spectra, along with DFT calculations of the surface segregation energy of Pd on the Cu single crystals and complementary scanning tunneling microscopy (STM) characterization, revealed the dynamic nature of segregation and alloying under reaction conditions, as well as their dependence on the environment and the crystallographic orientation of the surface.

STM images of Pd-decorated Cu(100), Cu(110), and Cu(111) single crystals acquired in UHV at room temperature immediately after Pd evaporation are shown in Figure 1. The initial surface morphology of the Pd/Cu samples differs significantly across the different crystallographic orientations. Upon Pd evaporation on Cu(100), the surface exhibits significant morphological roughness and is characterized by the formation of small islands, with no additional step-edge features (Figure 1(a)). These observations are consistent with the formation of a PdCu alloy on the Cu terraces, in agreement with past STM research on the nucleation and growth of Pd on Cu(100), where the formation of a c(2 × 2) PdCu alloy and the nucleation of the expelled Cu atoms into Cu islands were reported.^21^ The Pd/Cu(110) sample exhibits an even rougher surface morphology consistent with widespread PdCu alloying on the Cu terraces.^22^ Moreover, there are monolayer-deep pits (black structures in Figure 1(b)) where Cu atoms seem to have been expelled from the lattice. It has been suggested that these Cu atoms partly cover the PdCu alloy. Contrary to the other two crystallographic orientations, the Cu terraces on the Cu(111) surface are flat, with no significant roughening upon Pd evaporation (Figure 1(c)); the STM images suggest much less alloying. Instead, features consistent with Pd nucleation appear at the step edges and at defects on the large Cu terraces. Starting from the step edges, the Pd seems to form fingered islands on the terraces.^23^

**Figure 1:**
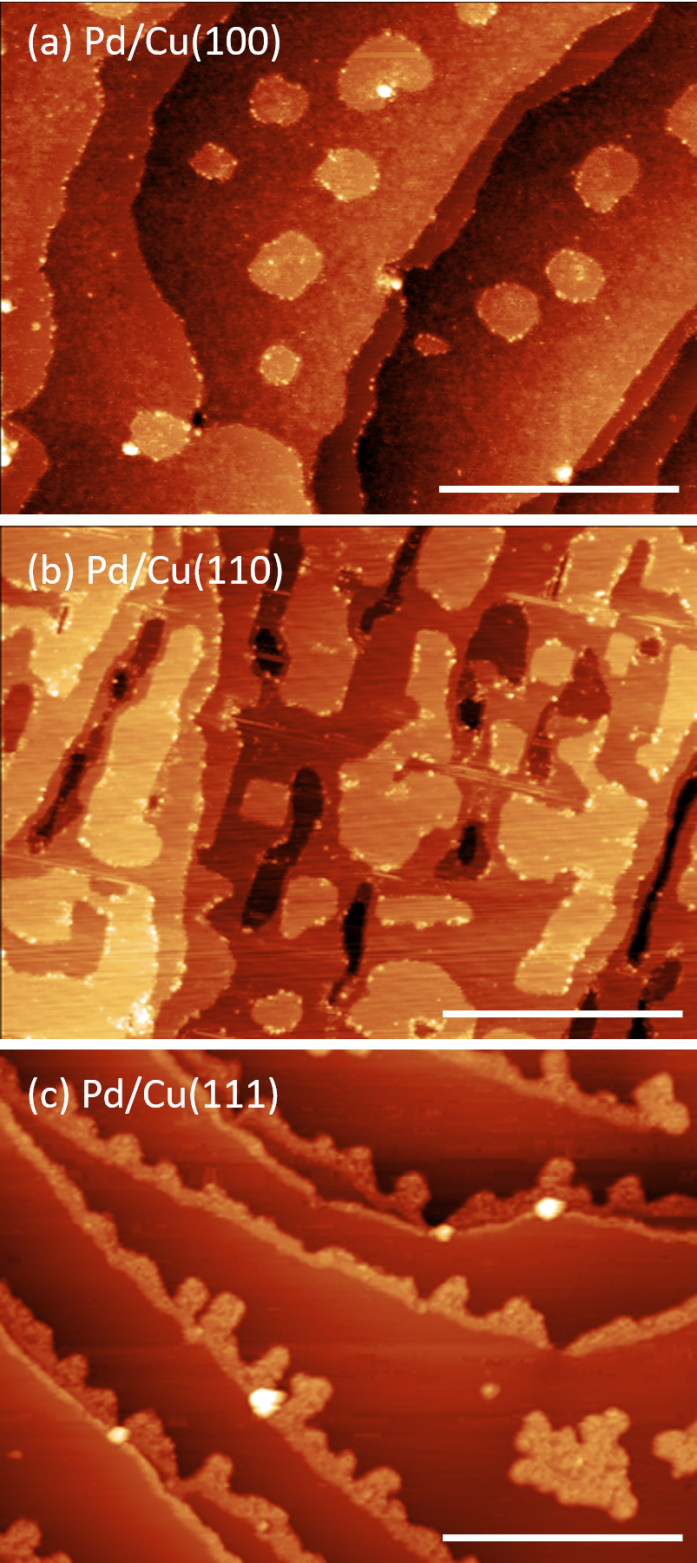
STM images of 0.2 ML Pd evaporated on (a) Cu(100) (tunneling voltage: *V*_t_ = −2 V; tunneling current: *I*_t_ = 200 pA), (b) Cu(110) (*V*_t_ = −1 V, *I*_t_ = 200 pA), and (c) Cu(111) (*V*_t_ = −1 V, *I*_t_ = 200 pA) single crystals at room temperature. All scale bars correspond to 50 nm.

XPS spectra from the Pd 3d core level of the 0.2 ML Pd islands evaporated on (a) Cu(100), (b) Cu(110), and (c) Cu(111) surfaces are shown in Figure 2. Additional Pd 3d spectra, obtained for reproducibility purposes, are shown in Figure S2. The XPS measurements were performed in vacuum at room temperature immediately after Pd evaporation and subsequently in the presence of 0.5 mbar H_2_ at 270 °C. Cu 2p XPS spectra of all three samples in UHV and in 0.5 mbar H_2_ are shown in Figure S3.

**Figure 2:**
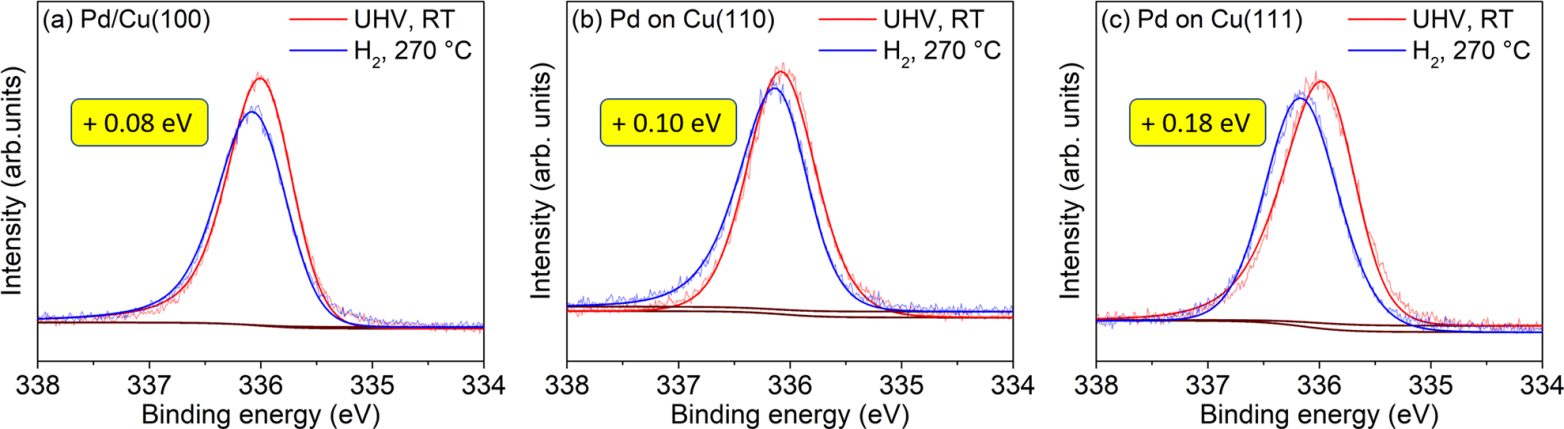
UHV-XPS (red) and NAP-XPS (blue) Pd 3d_5/2_ spectra of 0.2 ML Pd evaporated in UHV on (a) Cu(100), (b) Cu(110), and (c) Cu(111) single crystals. The NAP-XPS spectra were recorded in H_2_ (*P* = 0.5 mbar) at *T* = 270 °C. The yellow labels indicate the Pd 3d_5/2_ binding energy shift between the two environments.

It is known from previous studies that the Pd 3d_5/2_ binding energy can shift by up to 0.8 eV toward higher energies in Cu_*x*_Pd_1–*x*_ alloys due to partial electron transfer from Pd to Cu and subsequent changes in the Coulomb potential felt by the core electrons, as well as due to changes in the final state properties (hole state), involving the relaxation and the screening due to the surrounding medium.^24,25^ Therefore, the magnitude of the binding energy shift of the Pd 3d peak in a gaseous environment with respect to the UHV conditions can be used as an indicator of the extent of PdCu alloying. The higher the binding energy shift between UHV and the gaseous environment, the larger the difference with respect to the extent of PdCu alloying between vacuum and NAP-XPS conditions.

The Pd 3d binding energies determined from the spectra shown in Figure 2 and Figure S2 for all samples, together with the corresponding relative energy shifts Δ(BE), defined as Δ(BE) = BE_Gas_ – BE_UHV_, are shown in Table 1.

**Table 1:** Pd 3d_5/2_ Binding Energies of 0.2 ML Pd Evaporated on Cu(100), Cu(110), and Cu(111) Single Crystals Measured with XPS Directly after Evaporation in UHV and during Heating in H_2_ and in a CO_2_/H_2_ Mixture at 270°C

Pd 3d binding energy (eV)		Cu(100)	Cu(110)	Cu(111)
H_2_ 0.5 mbar	UHV, after evapor.	335.97 ± 0.02	336.08 ± 0.02	335.87 ± 0.04
	in gas, 270 °C	336.05 ± 0.02	336.18 ± 0.01	336.05 ± 0.04
	Δ(BE)^a^	**+**0.08 ± 0.02^b^	**+**0.10 ± 0.01	**+**0.18 ± 0.04
CO_2_ + H_2_ (1:3) 0.6 mbar	UHV, after evapor.	335.81 ± 0.02	335.86 ± 0.02	335.69 ± 0.01
	in gas, 270 °C	335.93 ± 0.03	336.00 ± 0.03	335.93 ± 0.01
	Δ(BE)	**+**0.12 ± 0.02	**+**0.14 ± 0.02	**+**0.24 ± 0.01

a The binding energy shift obtained by comparing the data in the gaseous atmosphere to those in UHV: Δ(BE) = BE_gas_ – BE_UHV_.

b For each environment and each crystallographic orientation, the average value of three independent measurements on three identical freshly prepared samples is given. The errors displayed are the standard deviation of three measurements per sample type.

It is evident from these results that the largest Pd 3d_5/2_ binding energy shift in H_2_ is observed on the Cu(111) surface ((0.18 ± 0.04) eV, Figure 2(c)). Within the error bar, Pd/Cu(100) and Pd/Cu(110) exhibit almost the same average shift [(0.08 ± 0.02) eV for Cu(100) (Figure 2(a)) and (0.10 ± 0.01) eV for Cu(110) (Figure 2(b))]. The positive energy shifts indicate that the extent of PdCu alloying increases in all samples upon heating in H_2_ as compared to the UHV conditions. The larger energy shift on Cu(111) reflects the larger relative change on this sample with respect to the UHV conditions. Cu(111) exhibits no or limited alloying in UHV, whereas some initial alloying is already present on Cu(100) and Cu(110) upon Pd evaporation, as indicated by the higher binding energies measured in UHV on the latter samples (Table 1). In the presence of H_2_, Pd/Cu(110) exhibits a higher Pd 3d_5/2_ binding energy (336.18 eV) than Pd/Cu(100) and Pd/Cu(111) (336.05 eV), indicating that Pd/Cu(110) has a higher amount of alloyed PdCu species. Thus, Pd/Cu(100) and Pd/Cu(111) seem to have similar final states in H_2_ with respect to alloying but exhibit significantly different binding energy shifts due to their different initial (UHV) states.

For the NAP-XPS studies under CO_2_ hydrogenation conditions, freshly prepared samples were investigated in vacuum after Pd evaporation and *in situ* under reaction conditions in a CO_2_ + H_2_ gas mixture (vol. 37/63, total pressure: 0.6 mbar) at 270 °C. The Pd 3d spectra of these samples are shown in Figure 3. Additional Pd 3d spectra, obtained for reproducibility purposes, are shown in Figure S4. Cu 2p XPS spectra of all three samples in UHV and in 0.6 mbar CO_2_ + H_2_ are depicted in Figure S5.

**Figure 3:**
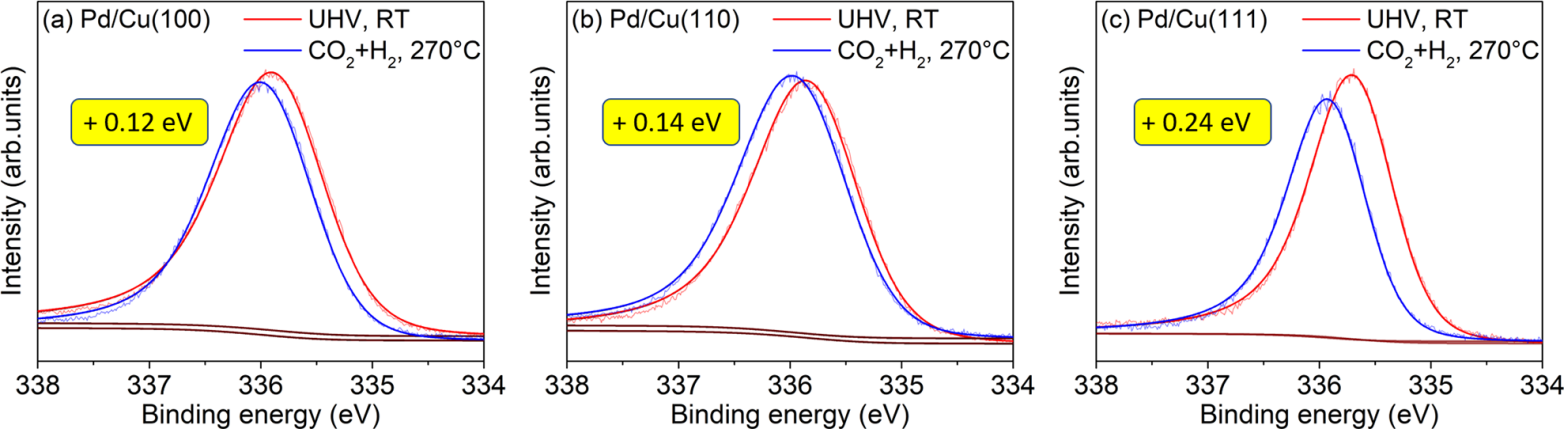
UHV-XPS (red) and NAP-XPS (blue) Pd 3d_5/2_ spectra of 0.2 ML Pd evaporated in UHV on (a) Cu(100), (b) Cu(110), and (c) Cu(111) single crystals at room temperature. The NAP-XPS spectra were recorded in a CO_2_ + H_2_ reaction mixture (vol % 1/3, *P* = 0.6 mbar) at *T* = 270 °C. The yellow labels indicate the binding energy shift of the Pd 3d_5/2_ main peak between the two environments.

Overall, the trends with regard to the binding energy shifts are similar to those observed in pure H_2_. The Pd/Cu(111) sample exhibits the largest binding energy shift of the Pd 3d_5/2_ peak ((0.24 ± 0.01) eV) under reaction conditions with respect to vacuum (Figure 3(c)). The Pd/Cu(100) and Pd/Cu(110) samples show smaller energy shifts of (0.12 ± 0.02) eV and (0.14 ± 0.02) eV, respectively (Figure 3(a) and Figure 3(b)). The amount of the relative shift is in all samples larger than in H_2_, indicating a stronger alloying effect under reaction conditions. In the reaction mixture, Pd/Cu(100) and Pd/Cu(111) have very similar binding energy values for the Pd 3d_5/2_ peak (335.93 eV), while on the Pd/Cu(110) surface, the peak is shifted to a higher value of 336.00 eV, indicating stronger alloying on the (110) surface.

In order to obtain understanding of the experimentally observed dependence of PdCu alloying on the crystallographic orientation of the Cu surface, DFT-RPBE calculations of the surface segregation energy of Pd atoms on Cu single crystalline surfaces were performed. The surface segregation energy determines whether the Pd atoms will tend to diffuse inward into the Cu lattice (indicated by positive segregation energy values), thus facilitating alloying, or whether they will tend to remain segregated on the surface, avoiding intermixing with Cu (indicated by negative values). Calculated surface segregation energies for Pd on Cu(100), Cu(110), and Cu(111) surfaces in vacuum and in the presence of various adsorbates are shown in Table 2.

**Table 2:** Surface Segregation Energy of Pd in Cu(100), Cu(110), and Cu(111) in the Presence of Various Reaction Educts and Intermediates Calculated by DFT-RPBE

Pd surface segregation energy (eV)		Cu(100)	Cu(110)	Cu(111)
UHV		0.19	0.73	–0.21
H_2_	H* (11%)^a^	0.03	0.59	–0.19
	H* (22%)	0.39	0.59 (H_2_*/0.82)	0.04
	H* (100%)	1.27	0.61	0.71
CH_3_O*	CH_3_O* (11%)	0.33	1.05	0.51
	CH_3_O* (22%)	1.13	1.73	1.56
HCOO*	HCOO* (11%)	0.34	1.04	0.28
	HCOO* (22%)	1.61	1.77	1.26
H_2_ (11%) + CO_2_ (11%) + CH_3_O* (11%)		0.41	1.16	0.52
H_2_ (11%) + CO_2_ (11%) + HCOO* (11%)		0.46	1.09	0.28

a The surface coverage of the gases, defined as the ratio of the number of adsorbates to the number of atoms in the top layer of the substrate, is shown in brackets.

In UHV, the segregation energy has a positive value for Cu(100) (0.19 eV) and an even larger positive value for Cu(110) (0.73 eV), whereas it is negative for Cu(111) (−0.21 eV). These values are qualitatively consistent with our results from STM (Figure 1) and UHV-XPS (Table 1), which both indicated significant PdCu alloying in vacuum on Cu(110), scarce alloying on Cu(111), and an intermediate situation with moderate alloying on Cu(100). Our calculations provide quantitative understanding of the crystallographic orientation dependence of the surface morphology of Pd-evaporated Cu surfaces first discussed based on STM data by Murray et al.^21,22^

In the presence of H_2_, the calculated segregation energies at moderate hydrogen coverage (22%) are positive for all crystallographic orientations of Cu, and thus, inward diffusion of Pd and Pd–Cu alloying is expected, in agreement with the experimental observations. For Cu(110), the segregation energy was calculated assuming the presence of molecularly adsorbed H_2_, because molecular adsorption was found to be more stable (by 0.07 eV) than dissociative adsorption on this surface. The opposite is the case on Cu(100) and Cu(111) surfaces, where dissociative adsorption is favored instead.^26^ The highest Pd surface segregation energy was calculated for Cu(110) (0.82 eV for molecularly adsorbed H_2_), consistent with the higher binding energy determined by XPS (336.18 eV). It is worth noting that Cu(110) would have the highest Pd segregation energy among all crystallographic orientations even if dissociative adsorption had been assumed instead (see Table 2). However, the high stability of molecular adsorption on Cu(110) further promotes the strong inward diffusion of Pd and its alloying with Cu. The influence of the crystallographic orientation on the relative stability of molecular adsorption versus that of dissociative adsorption, which is being reported here, should be taken into consideration in the ongoing discussion about the nature of hydrogen activation in hydrogenation reactions on Cu-based catalysts.^27−29^

The hydrogen coverage of 22% corresponds to a concentration of two H atoms per surface unit cell. While the thermodynamic estimate for the H coverage is only one H atom per surface unit cell, comparison of the calculated segregation energies with the XPS-derived binding energy values indicates that kinetic considerations need to be introduced to adjust the thermodynamically derived H coverage estimate. Aside from this fine-tuning, no further kinetic considerations are necessary, since the experimental findings regarding the extent of alloying are fully consistent with the thermodynamic calculations. It is noted that the formation of Pd hydrides is not expected to affect the core level binding energies measured by XPS in any significant way because the stability of hydrides is very low under the temperature and pressure conditions of our study considering that the H coverage is around 22% (Figure S15).

For the determination of the alloying and segregation trends under CO_2_ hydrogenation reaction conditions, it is important to consider the role of reaction intermediates too.^13^ Therefore, the segregation energy for Pd in Cu in the presence of the reaction intermediates CH_3_O* (methoxy) and formate (HCOO*) was also calculated. For Cu and Pd, HCOO* is more stable than CH_3_O*, since the formation energy of HCOO* is much lower than for the methoxy intermediate on all Pd/Cu surfaces (see Table S1). The formation energy differences are so large that taking into account realistic values of the chemical potentials for hydrogen and oxygen cannot change this conclusion (the free-energy differences do not depend on the chemical potential of CO_2_). Therefore, the segregation trend is expected to be dominated by the presence of the intermediate HCOO* on the surface. The presence of CO_2_ hardly affects the segregation trend due to its very weak adsorption. Like in pure H_2_, all segregation energies are positive, indicating inward diffusion of Pd and subsequent alloying. Under reaction conditions, the segregation energy is highest for Cu(110), in accordance with the high binding energy determined by XPS.

The DFT calculations of the surface segregation energy also explain why the relative binding energy shifts are larger in CO_2_ + H_2_ than in pure H_2_. The calculated segregation energies in the presence of the reaction gases and the stable intermediate HCOO* are higher (more positive) than in H_2_ on all surfaces. Thus, more alloying and therefore a higher shift in binding energy is expected for all crystallographic facets. Previous studies have attributed the improved activity of PdCu catalysts as compared to their monometallic Cu counterparts to the enhanced reducibility of the active Cu sites due to electron donation from Pd.^14^ Our study shows that surface alloying between Cu and Pd also needs to be taken into consideration as a factor that can influence the binding strength of the reactants and reaction intermediates.

In conclusion, it was shown that on model Pd/Cu catalysts the formation of a PdCu surface alloy in vacuum strongly depends on the crystallographic orientation of the Cu surface on which Pd is deposited due to the corresponding differences in the surface segregation energy. The latter has a high positive value on Cu(100) and Cu(110), facilitating inward Pd diffusion and PdCu alloying, whereas it is negative on Cu(111). In the presence of H_2_, as well as in the reaction mixture (CO_2_ + H_2_), alloying is promoted on all surfaces, with Pd/Cu(110) exhibiting the highest degree of PdCu alloying and Pd/Cu(111) showing the largest change. The differences in the segregation energy, and thus in the alloying trend, are partly due to differences in the mechanism of hydrogen adsorption, with molecular adsorption prevailing on Pd/Cu(110) and dissociative adsorption being favored on Pd/Cu(100) and Pd/Cu(111) instead. It was shown that the surface alloying during the CO_2_ hydrogenation reaction is driven predominantly by the stable reaction intermediate HCOO* and that the presence of CO_2_ hardly affects the surface morphology and composition. This observation underlines the role of reaction intermediates in the alloying and segregation behavior of bimetallic catalysts under reaction conditions.

## Supporting Information Available

Experimental details; details of XPS analysis and binding energy calibration; additional XPS analysis results; XPS reproducibility measurements; additional STM and XPS data for lower Pd coverages; and details of DFT calculations (PDF)
