# Supplementary material for: Crystallographic Orientation Dependence of Surface Segregation and Alloying on PdCu Catalysts for CO2 Hydrogenation
Source: J Phys Chem Lett. 2021 Mar 9;12(10):2570–5. doi: 10.1021/acs.jpclett.1c00179 (PMC7983046; doi:10.1021/acs.jpclett.1c00179)
Supplement: Supplementary file 1 — jz1c00179_si_001.pdf [file jz1c00179_si_001.pdf]

Supporting information

Crystallographic Orientation Dependence of Surface Segregation and Alloying on PdCu Catalysts for CO_2 Hydrogenation

Lukas Pielsticker[†,∥], *Ioannis Zegkinoglou*[†], *Zhong-Kang Han*[‡], *Juan J. Navarro*[§], *Sebastian Kunze*[§], *Osman Karslıoğlu*[§], *Sergey V. Levchenko*[‡], *and Beatriz Roldan Cuenya*[§,*]

[†]Faculty of Physics and Astronomy, Ruhr University Bochum, 44780 Bochum, Germany

[‡]Center for Energy Science and Technology, Skolkovo Institute of Science and Technology, Moscow 121205, Russia

[§]Department of Interface Science, Fritz-Haber Institute of the Max Planck Society, Berlin 14195, Germany

Corresponding Author

* Roldan@fhi-berlin.mpg.de

Present Addresses

[∥]Department Heterogeneous Reactions, Max Planck Institute for Chemical Energy Conversion, Mülheim a.d. Ruhr, Germany

Experimental details

Pd nanoislands were grown by electron beam evaporation on clean Cu single crystals of different crystallographic orientations (Pi-Kem Ltd., purity: 99.999) inside the preparation chamber of a NAP-XPS ultrahigh vacuum (UHV) system. The preparation chamber is equipped with an ion source for sputter cleaning, an electron beam heater for sample annealing and a single pocket electron beam evaporator for the growth of metallic thin films on substrates. The electron beam evaporator was loaded with a Pd rod (Goodfellow GmbH, Ø 2 mm, purity: 99.95 %). The ion source was connected through a leak-valve to an Ar lecture bottle (Linde GmbH, purity 5.0). Cu(100), Cu(110), and Cu(111) single crystals were loaded on flat stainless-steel sample holders. The crystals were treated following a protocol that involved three consecutive cycles of Ar sputtering ($P = 3.0 \cdot 10^{-5}$ mbar, $E = 2.5$ keV, duration: 45 min per cycle) and subsequent annealing in vacuum at 520 °C (45 min during the first and second cycles; 90 min during the third cycle). The absence of contamination on the Cu single crystals after the sputtering and annealing treatment was systematically verified by XPS. Survey, C 1s and Pd 3d spectra of a Cu(110) single crystal after sputtering and annealing are exemplarily shown in Figure S1.

After the surface treatment, Pd was deposited onto the Cu single crystals by electron beam evaporation. The deposition rate of Pd was controlled with a quartz microbalance connected to the preparation chamber and amounted to approximately 1 monolayer (ML)/220 s = 0.27 ML/min when the Pd flux current was kept constant at 74.0 nA. The chosen evaporation time for all samples was 52 s, resulting in a nominal Pd coverage of 0.2 MLs. Samples exposed to shorter (15 s) and longer (3 min) Pd evaporation, thus exhibiting lower and higher Pd coverage, respectively, were also investigated for comparison, in order to examine a possible effect of the Pd coverage on the alloying and segregation behavior.

A second set of Pd/Cu samples was prepared following the same procedure as described above in a separate, identically equipped preparation chamber to be used for morphological characterization in an STM characterization setup (Aarhus 150, Specs GmbH). Identical Cu crystals, substrate cleaning and Pd evaporation protocols as for the XPS samples were employed.

The analysis chamber of the NAP-XPS setup is equipped with a differentially pumped PHOIBOS 150 Near Ambient Pressure Hemispherical Energy Analyzer and a monochromatized Al K_α X-ray source (1486.7 eV). XPS spectra of the Pd-covered Cu crystals were obtained in UHV at room temperature (after Pd evaporation), in pure H_2 (0.6 mbar) and under CO_2 hydrogenation conditions (volume ratio CO_2:H_2 : 37:63, total pressure: 0.6 mbar). The pressure was regulated by dosing the constituent gases through separate leak valves into the main analysis chamber. The sample temperature during the NAP-XPS measurements was kept constant at 270 °C with an infrared heater (halogen lamp) located on the sample manipulator. After each measurement, the sample was cooled down in the gas and the chamber was evacuated when the sample reached room temperature. For each one of the three different crystallographic orientations of Cu, the XPS study was repeated three times for the CO_2 hydrogenation conditions as well as for the pure H_2 environment, each time on a freshly prepared sample to ensure the reproducibility of the results.

Details of the XPS analysis and binding energy calibration

For all Pd spectra, the metallic Cu $2p_{3/2}$ peak was used as binding energy reference. While some of the Cu atoms are also expected to be present in the alloyed state, it was assumed that the main contribution to the Cu XPS signal is from the unalloyed component, especially considering the small Pd coverage that was discovered for these samples with STM (see Fig. 1 in the main text). Therefore, all Cu $2p_{3/2}$ peaks were aligned at 932.6 eV and used as energy reference for all other peaks.

In agreement with the available literature, Cu was fitted with a doublet for the $2p_{3/2}$ and $2p_{1/2}$ regions, with the single peak of the Cu $2p_{1/2}$ region at 932.6 eV.[1] The fits were constrained with respect to the area ratio (1:2) and the binding energy offset in virtue of the spin-orbit splitting (19.8 eV for metallic Cu in Cu 2p spectra). The two metallic Cu peaks were fitted with Gaussian-Lorentzian line shapes (ratio 0.9) and a Shirley background. For the Pd 3d spectra, a metallic doublet with an asymmetric line-shape was used in the fittings.[2] The area ratio (2:3) and the doublet energy splitting of 5.26 eV were constrained in the fitting. In order to account for initial alloying and final state effects, the binding energy of the Pd $3d_{5/2}$ was allowed to vary by ± 0.4 eV compared to the literature value. The asymmetry parameter, the full width at half maximum (FWHM) and the ratio of the Gaussian and Lorentzian portion of the line shapes were also allowed to vary. The fits were adjusted by minimizing the residual of the fits with respect to the measured spectra.

It should be noted that both the standard deviation error of the measurements (0.01 eV) and the nominal energy resolution of the spectrometer at the Al Kα line (about 0.008 eV) are at least one order of magnitude smaller than the reported binding energy shifts. Therefore, the conclusions reported in the main text regarding the alloying trends are beyond experimental uncertainties.

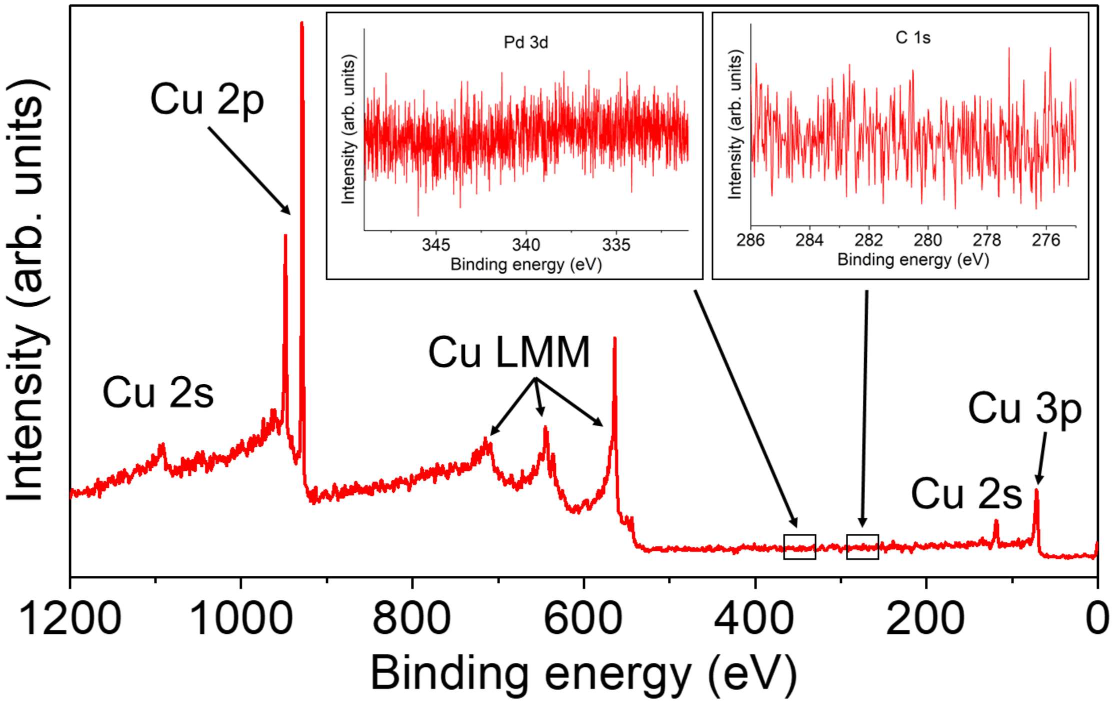

Figure S1. XPS survey of a Cu(110) single crystal after repeated cycles of Ar sputtering and annealing. The inset shows high-resolution scans of the C 1s and Pd 3d regions. The survey spectra only exhibit peaks corresponding to Cu, namely the Cu 2s, Cu 2p, Cu 3s and Cu 3p photoemission peaks and the Cu LMM Auger electron peak, thus demonstrating the cleanliness and the lack of impurities on the surface after the preparation process. No O 1s or C 1s contributions are observed.

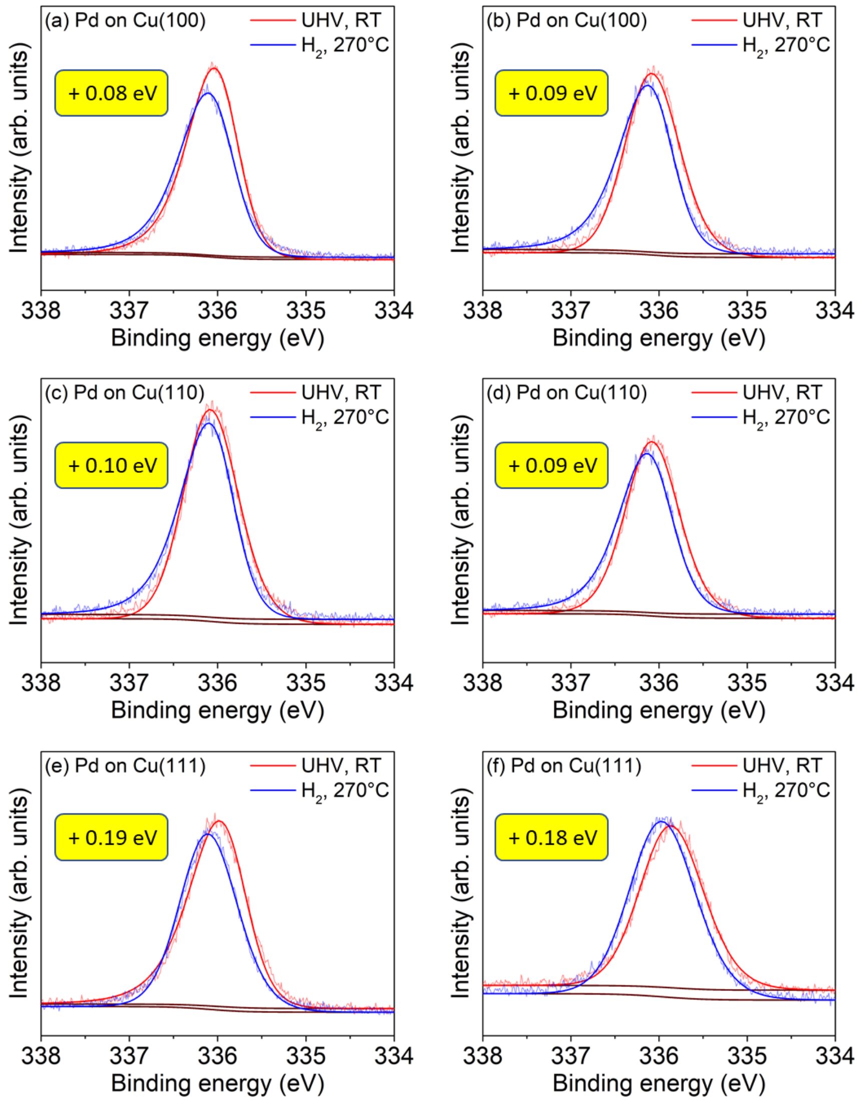

Figure S2. Pd $3d_{5/2}$ XPS spectra additional to those shown in Figure 2 measured in UHV (red) and in H_2 (0.5 mbar, 270 °C) (blue) on Pd-evaporated (a, b) Cu(100), (c, d) Cu(110), and (e, f) Cu(111) single crystals (coverage: 0.2 ML). The labels in yellow indicate the binding energy shift of the Pd $3d_{5/2}$ main peak between the spectra in UHV and those in H_2. These data correspond to new fresh samples identically prepared as the ones shown in the main text.

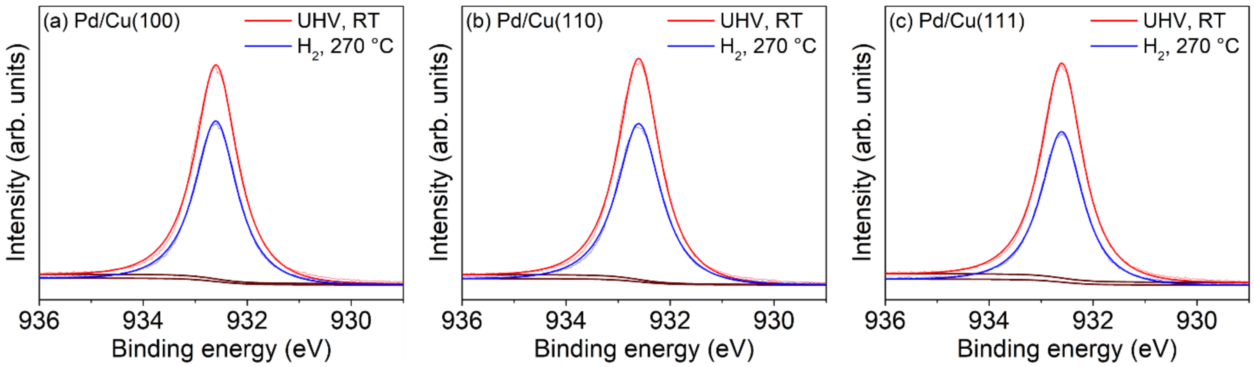

Figure S3. UHV-XPS (red) and NAP-XPS (blue) Cu $2p_{3/2}$ spectra of Pd-decorated (a) Cu(100), (b) Cu(110), and (c) Cu(111) single crystals. All NAP-XPS spectra were recorded in pure H_2 gas at P = 0.5 mbar and T = 270 °C.

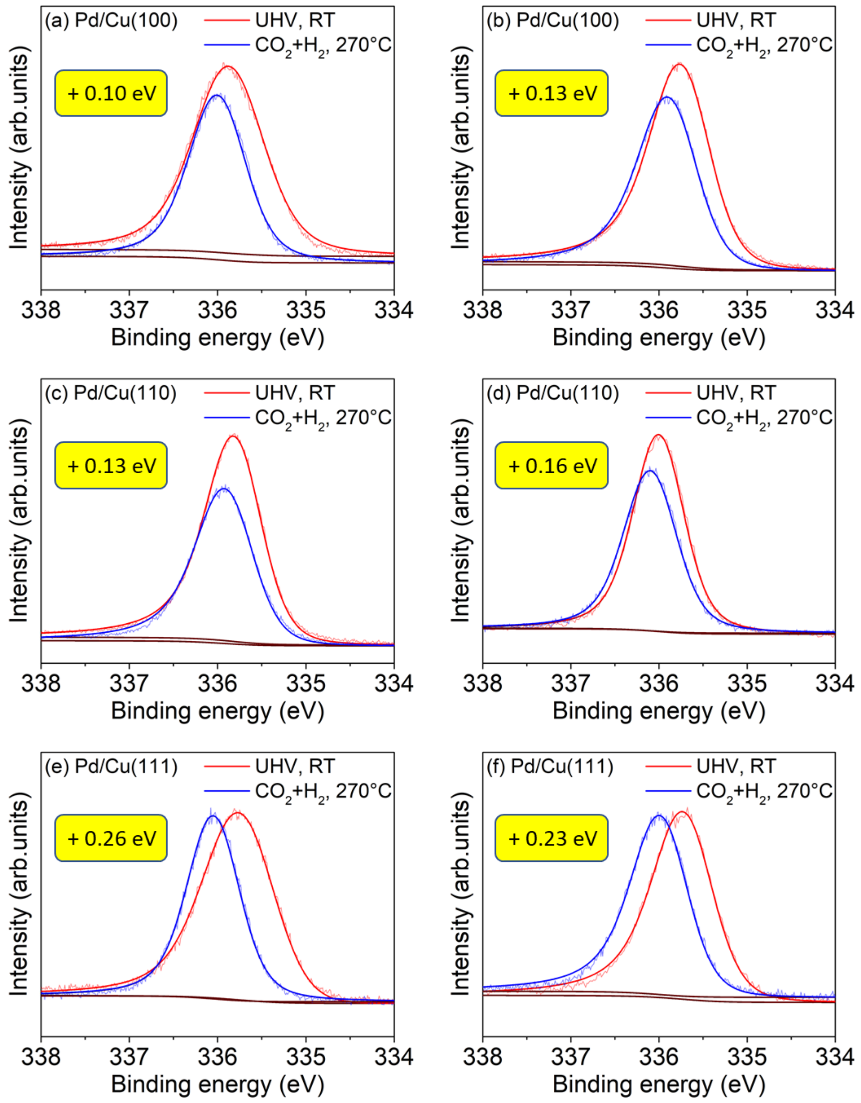

Figure S4. Pd $3d_{5/2}$ XPS spectra additional to those shown in Figure 3 measured in UHV (red) and in CO_2 + H_2 (0.6 mbar, 270 °C) (blue) on Pd-evaporated (a, b) Cu(100), (c, d) Cu(110), and (e, f) Cu(111) single crystals (coverage: 0.2 ML). The labels in yellow indicate the binding energy shift of the Pd $3d_{5/2}$ main peak between the spectra in UHV and those in CO_2 + H_2. The variation of the binding energy shift (standard deviation error) was determined to be 0.01 – 0.02 eV among different measurements. These data correspond to new fresh samples identically prepared as the ones shown in the main text.

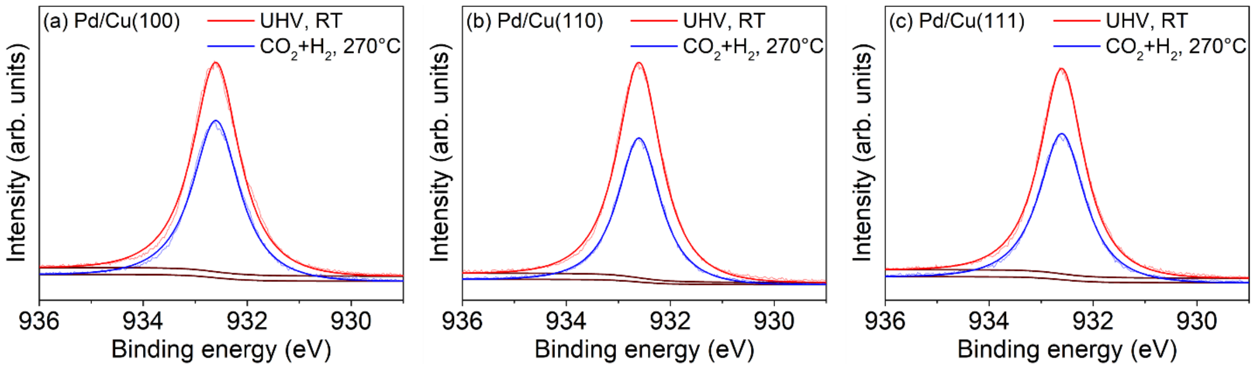

Figure S5. UHV-XPS (red) and NAP-XPS (blue) Cu $2p_{3/2}$ spectra of Pd-decorated (a) Cu(100), (b) Cu(110), and (c) Cu(111) single crystals. All NAP-XPS spectra were recorded in a CO_2 + H_2 reaction mixture at P = 0.6 mbar and T = 270 °C.

Effect of the Pd surface coverage

In the main text, the Pd $3d_{5/2}$ energy shift is used as an indicator for the alloying of Pd and Cu after segregation of the materials. In order to confirm this assumption, additional tests with different Pd coverages were performed.

An STM image of Pd evaporated with a lower coverage on a Cu(111) single crystal is shown in Figure S6. The lower coverage was achieved by decreasing the evaporation time from 52.0 s to 15.0 s while keeping the Pd flux of the evaporator constant. The comparison of Figures 1(c) and S6 clearly demonstrates that the surfaces look very similar after the evaporation and that the only difference is the decreased surface coverage of Pd. Again, Pd decorates the step edges and defects of the Cu(111) surface, with no initial alloy formation observed.

Figure S7 shows UHV- and NAP-XPS spectra of Cu(100), Cu(110) and Cu(111) samples that were prepared identically to that of Figure S6. The Cu:Pd ratio was 96:4, as determined by fitting of the XPS peaks. The binding energy shifts between UHV and the CO_2 + H_2 reaction mixture of 0.12 eV on Cu(100), 0.14 eV on Cu(110) and 0.23 eV on Cu(111) qualitatively confirm that the strongest alloying of Pd and Cu can be observed on Cu(111), followed by Cu(110) and Cu(100). Interestingly, the binding energy shifts measured on the samples with lower coverage are all within the experimentally determined error bars of the shift observed on the samples which contained more Pd. Evidently, decreasing the Pd coverage (by ~2/3 of the original amount) does not affect the trends in the Pd-Cu samples.

Additional measurements were also performed on a Pd/Cu(111) sample with a higher coverage of 0.7 ML. Figure S8 shows UHV and NAP-XPS spectra of this Pd/Cu(111) sample. By fitting of the XPS peaks, a Cu:Pd elemental ratio of 79:21 was determined. The binding energy shift of 0.24 eV is also within the experimentally determined error bars of the shift observed on the samples with evaporation times of 52 s (0.2 ML coverage).

These experiments with lower and higher coverages further support the conclusion that the observed shift in binding energy can be explained by segregation and alloying on the individual Pd nanoislands.

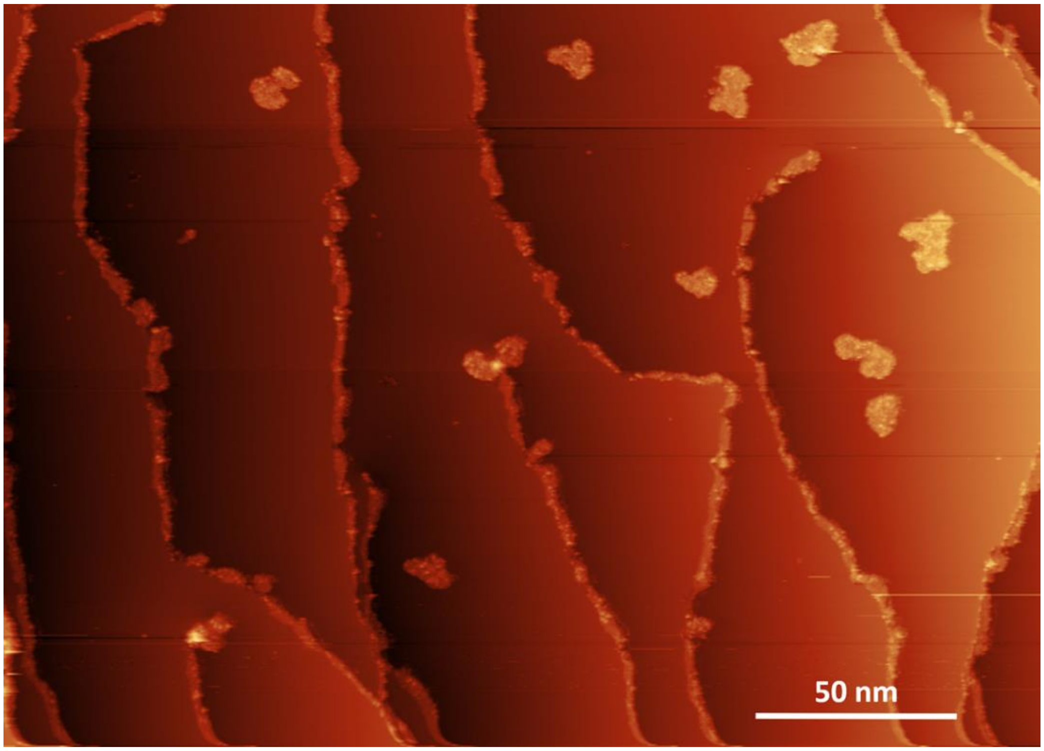

Figure S6. STM image of Pd islands evaporated on a Cu(111) single crystal with a total coverage of 0.05 ML.

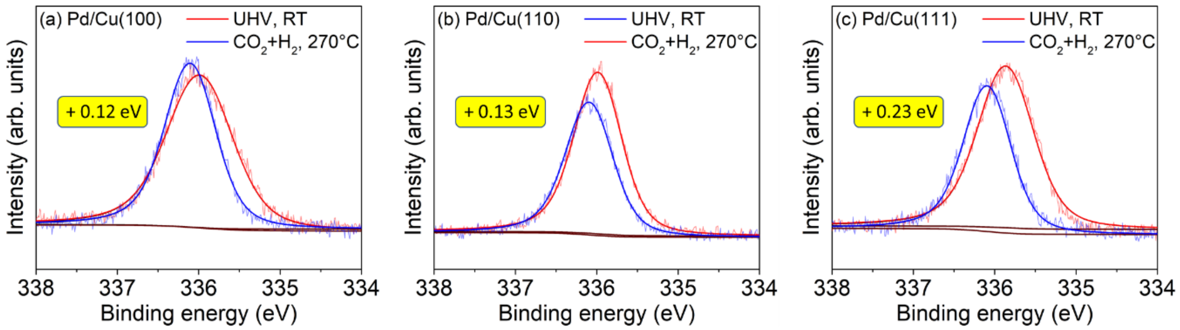

Figure S7. UHV-XPS (red) and NAP-XPS (blue) Pd 3d spectra of 0.05 ML Pd evaporated in UHV on (a) Cu(100), (b) Cu(110) and (c) Cu(111) single crystals. The NAP-XPS spectra were recorded in a CO_2 + H_2 reaction mixture at P = 0.6 mbar and T = 270 °C. The yellow labels indicate the binding energy shift of the Pd $3d_{5/2}$ main peak between the measurements in UHV and those under CO_2 hydrogenation reaction conditions.

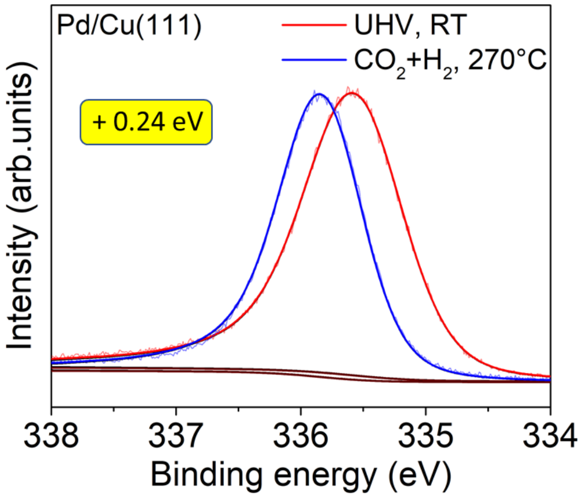

Figure S8. UHV-XPS (red) and NAP-XPS (blue) Pd 3d spectra of 0.7 ML Pd evaporated in UHV on (a) Cu(100), (b) Cu(110) and (c) Cu(111) single crystals. The NAP-XPS spectrum was recorded in a CO_2 + H_2 reaction mixture at P = 0.6 mbar and T = 270 °C. The yellow labels indicate the binding energy shift of the $Pd3d_{5/2}$ main peak between the measurements in UHV and those under CO_2 hydrogenation reaction conditions.

Table S1. Formation energies of CH_3O and HCOO on Pd-covered Cu(100), Cu(110), and Cu(111) single crystals. The formation energy for HCOO is calculated as $E_f(HCOO) = E(HCOO+metal) - E(metal) - E(CO_2) - 1/2\ E(H_2)$, while the formation energy for CH_3O is calculated by $E_f(CH_3O) = E(CH_3O+metal) - E(metal) - E(CO_2) + 1/2\ E(O_2) - 3/2\ E(H_2)$.

Formation energy (eV)	HCOO	CH_3O
Pd/Cu(100)	-0.30	1.81
Pd/Cu(110)	-0.51	1.64
Pd/Cu(111)	-0.06	1.86

Computational details

All *ab initio* calculations were performed with the revised Perdew-Burke-Ernzerhof (RPBE) functional[3] as implemented in the all-electron full-potential electronic-structure code FHI-aims[4] using numerical atom-centered basis functions. The standard 'tight' settings (grids and basis functions) were employed. A 3×3 supercell slab with eleven metal layers was considered, with the two bottom layers fixed and the rest of the layers fully relaxed until the maximum remaining force fell below 10^{-2} eV/Å. A 5×5×1 *k*-point grid was employed. This ensures the convergence of the segregation energies and adsorption energies within 0.05 eV. The adsorbates were placed on one side of the slab and a dipole correction was applied. The lattice vector along the direction parallel to the vacuum gap was 50 Å.

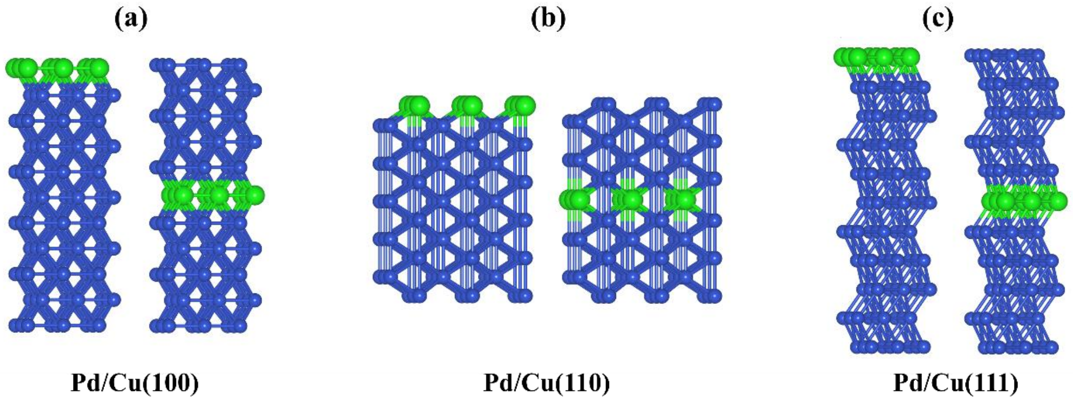

Figure S9. Structure models of surface overlayer and bulk alloy Pd in (a) Cu(100), (b) Cu(110), and (c) Cu(111) crystals. Cu and Pd atoms are shown as blue and green spheres, respectively.

The surface segregation energy, SE, is defined as the energy difference when moving the Pd monolayer from the bulk to the surface (Figure S9) in vacuum or when an adsorbate is present (the adsorbate is assumed at the most stable adsorption site for each system). In this work, the surface segregation energy is calculated using equation (1), where E_{surface} and $E_{n\text{th-layer}}$ correspond to the total RPBE energy of the slab with the Pd monolayer on the top and the nth surface layer, respectively. The value of n is chosen so that the energy difference between $E_{n\text{th-layer}}$ and $E_{(n\text{-}1)\text{th-layer}}$ is less than 0.05 eV.

$$\text{SE} = E_{\text{surface}} - E_{n\text{th-layer}} \qquad (1)$$

The calculated most stable configurations for Pd/Cu systems with different H coverages are displayed in Figure S10. The hydrogen coverage is calculated as the ratio between the total number of H adatoms and the number of atoms in one atomic layer. Using this definition, the range of H concentration considered is 11% - 100%, with the lower limit corresponding to one H atom and the upper limit to 9 H (1 ML H) atoms in the 3×3 cell.

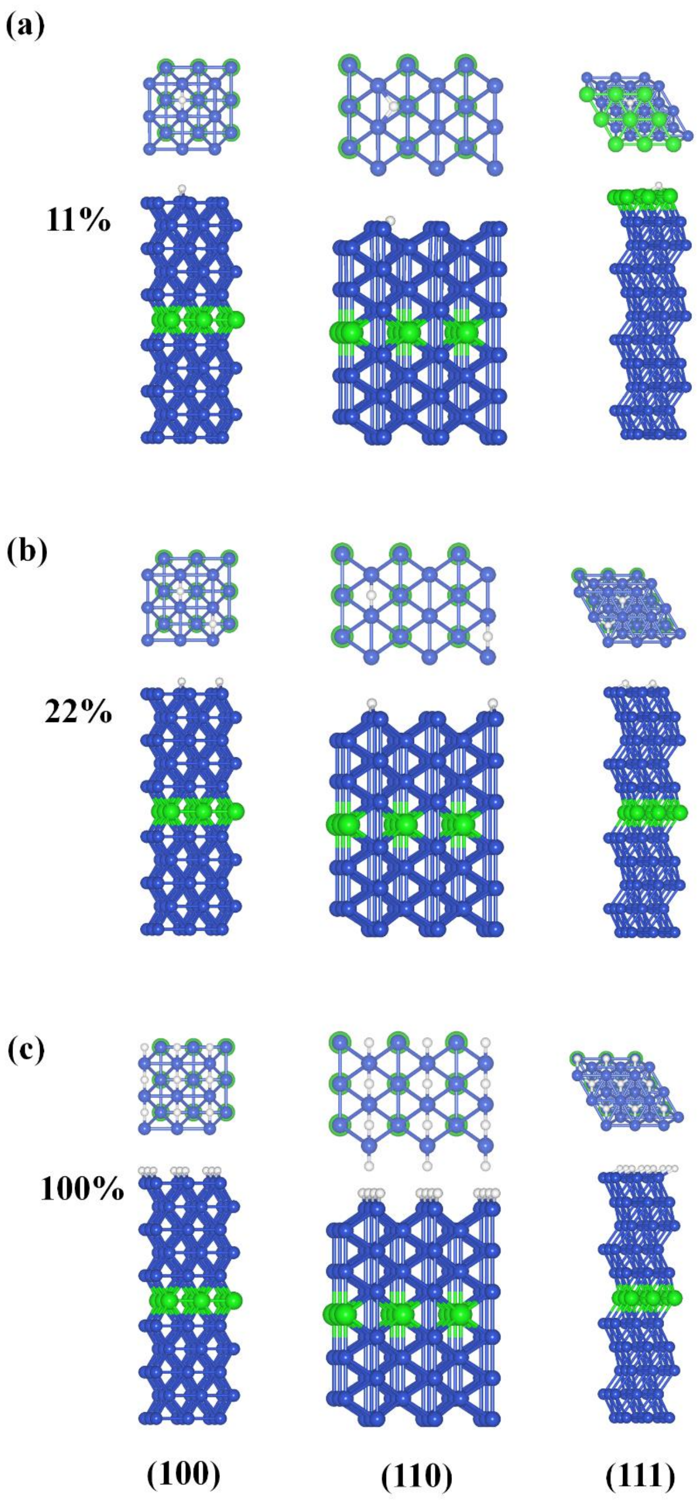

Figure S10. The most stable configurations for Pd/Cu systems with different H coverages. The figures show surface supercells for each surface cut. Cu, Pd, and H atoms are shown as blue, green, and light grey spheres, respectively.

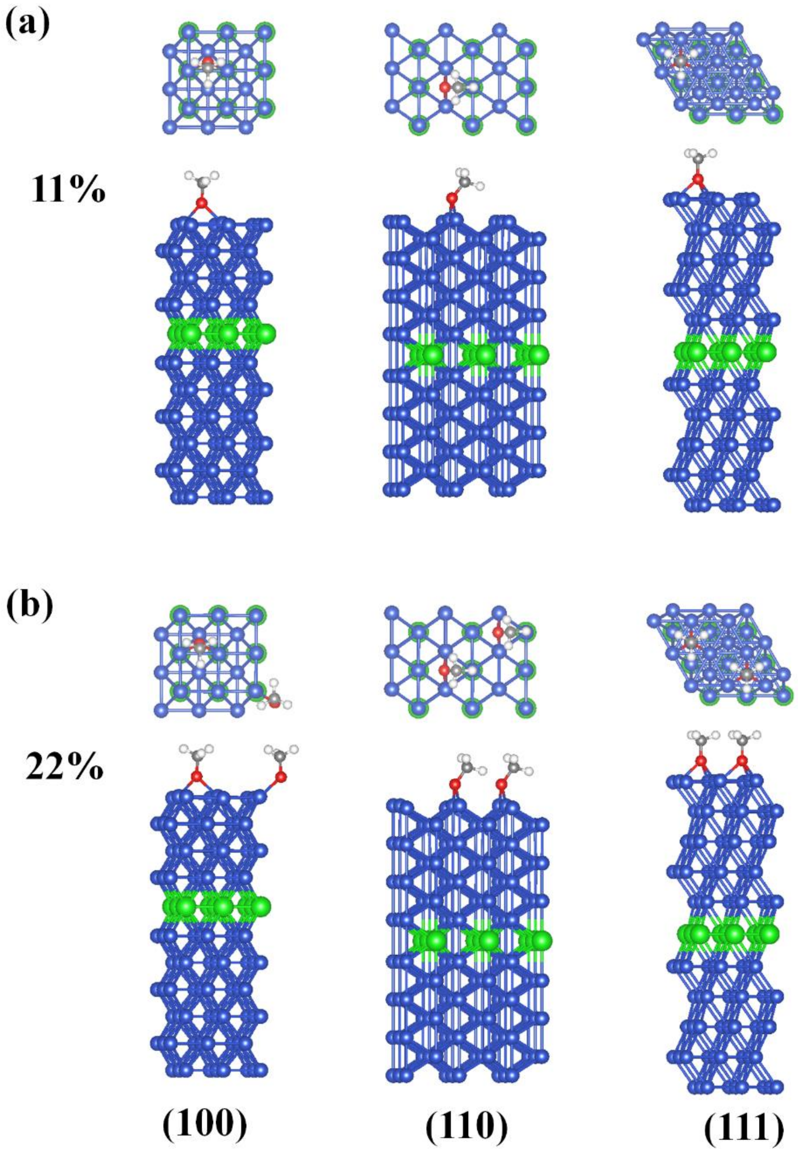

Figure S11. The most stable configurations for Pd/Cu systems with different CH_3O coverages. The figures show surface supercells for each surface cut. Cu, Pd, O, C, and H atoms are shown as blue, green, red, grey, and light grey spheres, respectively.

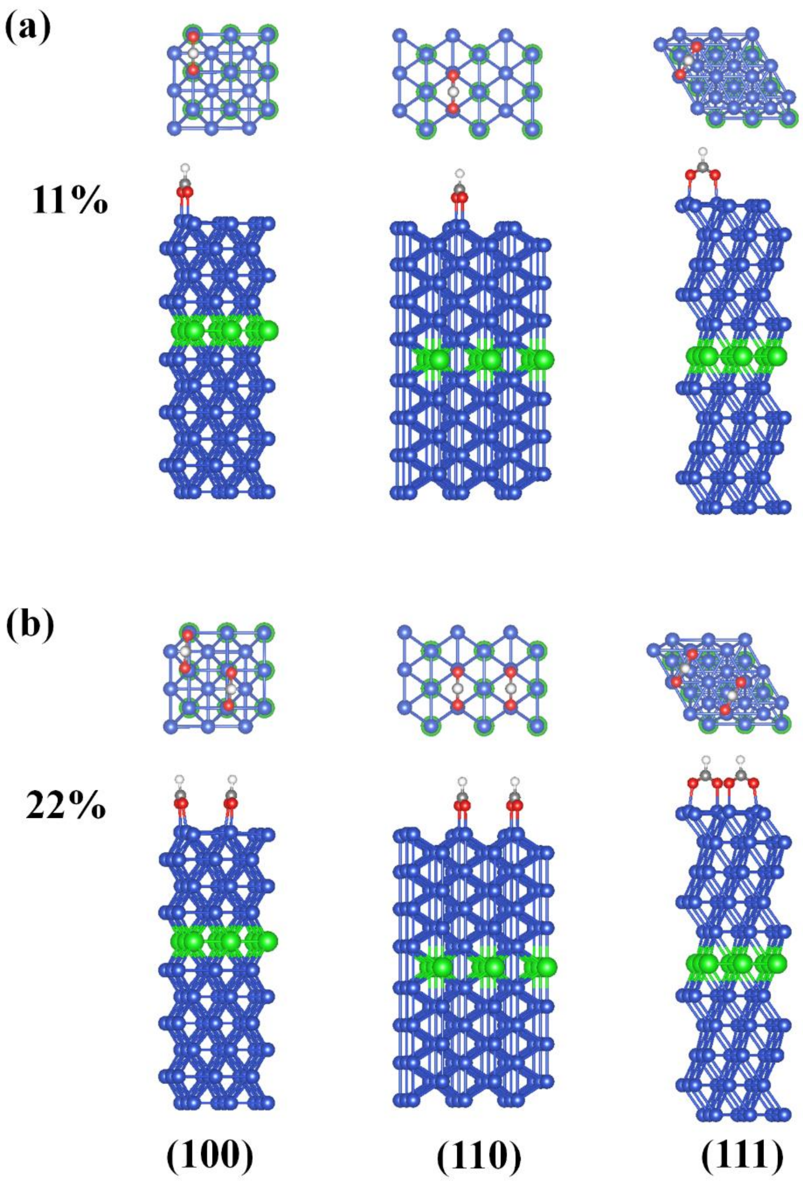

Figure S12. The most stable configurations for Pd/Cu systems with different HCOO coverages. The figures show surface supercells for each surface cut. Cu, Pd, O, C, and H atoms are shown as blue, green, red, grey, and light grey spheres, respectively.

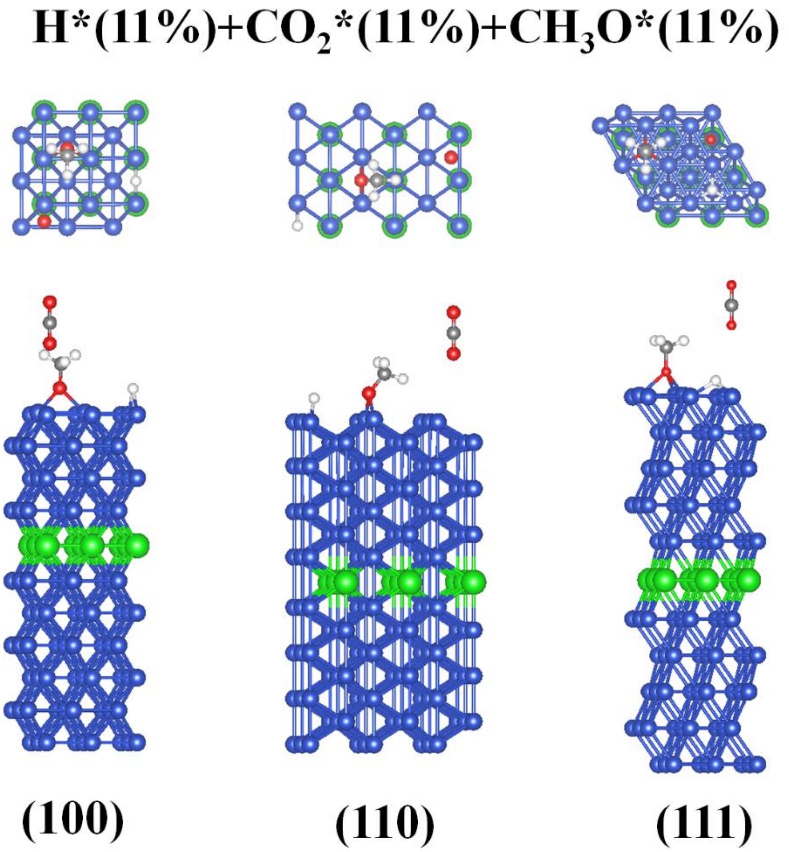

Figure S13. The most stable configurations for Pd/Cu systems with co-adsorbed H, CO_2, and CH_3O. The figures show surface supercells for each surface cut. The coverage is 11% for each molecule. Cu, Pd, O, C, and H atoms are shown as blue, green, red, grey, and light grey spheres, respectively.

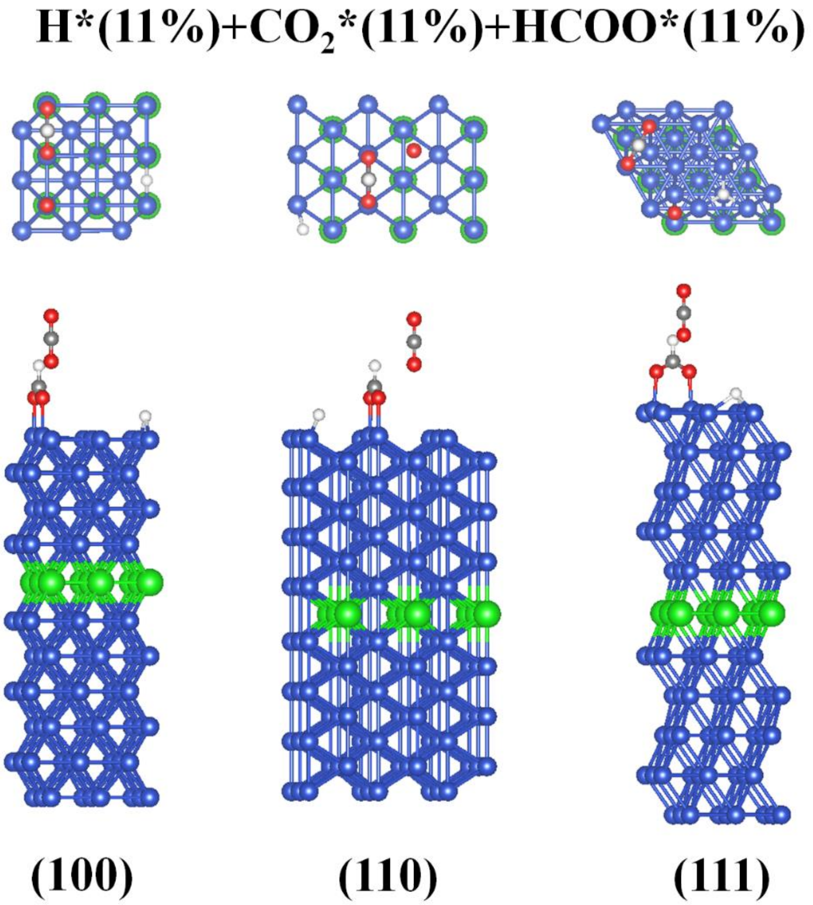

Figure S14. The most stable configurations for Pd/Cu systems with co-adsorbed H, CO_2, and HCOO. The figures show surface supercells for each surface cut. The coverage is 11% for each molecule. Cu, Pd, O, C, and H atoms are shown as blue, green, red, grey, and light grey spheres, respectively.

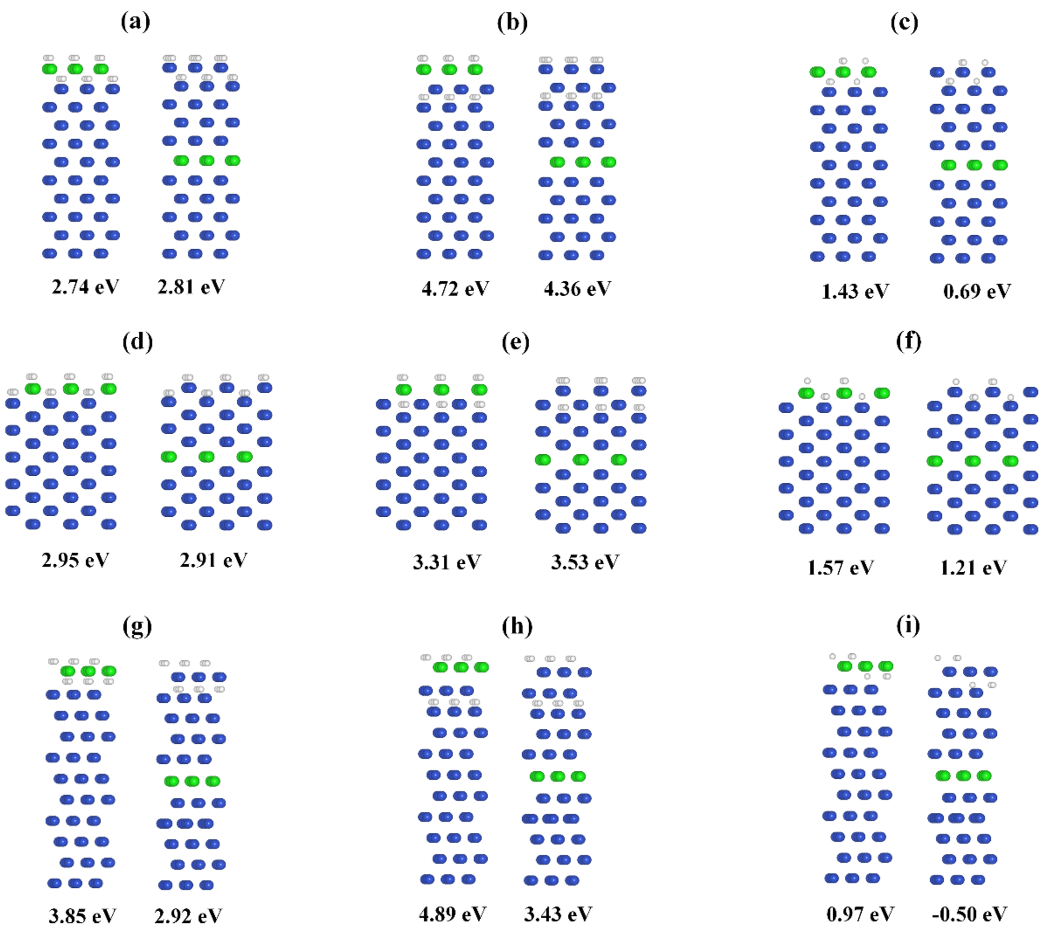

Figure S15. Hydride formation energy for various Pd hydride structures on (a-c) Pd/Cu(100), (d-f) Pd/Cu(110), and (g-i) Pd/Cu(111) systems. The hydride formation energy was calculated according to: E_f (hydride) = E(total) – E(slab) – 0.5N*E(H_2), where E(total), E(slab), and E(H_2) are the energy of the total system, the energy of the surface slab, and the energy of the hydrogen molecule, respectively. N is the number of hydrogen adatoms.

REFERENCES

(1) Biesinger, M. C.; Lau, L. W. M.; Gerson, A. R.; Smart, R. S. C. Resolving Surface Chemical States in XPS Analysis of First Row Transition Metals, Oxides and Hydroxides: Sc, Ti, V, Cu and Zn. *Applied Surface Science* **2010,** *257* (3), 887–898.
(2) Gabasch, H.; Unterberger, W.; Hayek, K.; Klötzer, B.; Kleimenov, E.; Teschner, D.; Zafeiratos, S.; Hävecker, M.; Knop-Gericke, A.; Schlögl, R. In Situ XPS Study of Pd (1 1 1) Oxidation at Elevated Pressure, Part 2: Palladium Oxidation in the 10−1 Mbar Range. *Surface Science* **2006,** *600* (15), 2980-2989.
(3) Hammer, B.; Hansen, L. B.; Nørskov, J. K. Improved Adsorption Energetics within Density-Functional Theory Using Revised Perdew-Burke-Ernzerhof Functionals. *Physical Review B* **1999,** *59* (11), 7413.
(4) Blum, V.; Gehrke, R.; Hanke, F.; Havu, P.; Havu, V.; Ren, X.; Reuter, K.; Scheffler, M. Ab Initio Molecular Simulations with Numeric Atom-Centered Orbitals. *Computer Physics Communications* **2009,** *180* (11), 2175-2196.
